# Multilevel comparative bioinformatics to investigate evolutionary relationships and specificities in gene annotations: an example for tomato and grapevine

**DOI:** 10.1186/s12859-018-2420-y

**Published:** 2018-11-30

**Authors:** Luca Ambrosino, Valentino Ruggieri, Hamed Bostan, Marco Miralto, Nicola Vitulo, Mohamed Zouine, Amalia Barone, Mondher Bouzayen, Luigi Frusciante, Mario Pezzotti, Giorgio Valle, Maria Luisa Chiusano

**Affiliations:** 1 Department of Agriculture, University of Naples “Federico II,”, Portici, Naples, Italy; 2 Department of Biotechnology, University of Verona, Verona, Italy; 3 Génomique et Biotechnologie des Fruits, UMR990 INRA / INP-Toulouse, Université de Toulouse, Castanet-Tolosan, France; 4 CRIBI Biotechnology Centre, University of Padova, Padova, Italy; 5 Research Infrastructures for Marine Biological Resources, Stazione Zoologica Anton Dohrn, Naples, Italy; 6 Current address: Research Infrastructures for Marine Biological Resources, Stazione Zoologica Anton Dohrn, Naples, Italy; 7 Current address: Center for Research in Agricultural Genomics, Cerdanyola, Barcelona, Spain; 8 Current address: Plants for Human Health Institute, North Carolina State University, Kannapolis, NC USA

**Keywords:** Comparative genomics, Orthologs, Paralogs, Tomato, Grapevine, Species specific gene loci

## Abstract

**Background:**

“Omics” approaches may provide useful information for a deeper understanding of speciation events, diversification and function innovation. This can be achieved by investigating the molecular similarities at sequence level between species, allowing the definition of ortholog and paralog genes. However, the spreading of sequenced genome, often endowed with still preliminary annotations, requires suitable bioinformatics to be appropriately exploited in this framework.

**Results:**

We presented here a multilevel comparative approach to investigate on genome evolutionary relationships and peculiarities of two fleshy fruit species of relevant agronomic interest, *Solanum lycopersicum* (tomato) and *Vitis vinifera* (grapevine). We defined 17,823 orthology relationships between tomato and grapevine reference gene annotations. The resulting orthologs are associated with the detected paralogs in each species, permitting the definition of gene networks, useful to investigate the different relationships. The reconciliation of the compared collections in terms of an updating of the functional descriptions was also exploited. All the results were made accessible in ComParaLogs, a dedicated bioinformatics platform available at http://biosrv.cab.unina.it/comparalogs/gene/search.

**Conclusions:**

The aim of the work was to suggest a reliable approach to detect all similarities of gene loci between two species based on the integration of results from different levels of information, such as the gene, the transcript and the protein sequences, overcoming possible limits due to exclusive protein versus protein comparisons. This to define reliable ortholog and paralog genes, as well as species specific gene loci in the two species, overcoming limits due to the possible draft nature of preliminary gene annotations. Moreover, reconciled functional descriptions, as well as common or peculiar enzymatic classes and protein domains from tomato and grapevine, together with the definition of species-specific gene sets after the pairwise comparisons, contributed a comprehensive set of information useful to comparatively exploit the two species gene annotations and investigate on differences between species with climacteric and non-climacteric fruits. In addition, the definition of networks of ortholog genes and of associated paralogs, and the organization of web-based interfaces for the exploration of the results, defined a friendly computational bench-work in support of comparative analyses between two species.

**Electronic supplementary material:**

The online version of this article (10.1186/s12859-018-2420-y) contains supplementary material, which is available to authorized users.

## Background

The detection of differences and/or similarities in genome organization among different species is a key approach in comparative genomics. It helps the understanding of common aspects or distinctive peculiarities and also the transfer of information (e.g. annotated features) from one species to another, gathering evidence to infer the main evolutionary mechanisms that shaped the molecular structure and the functionality of the genomes of the investigated species [1, 2].

Most computational methods in comparative genomics are based on sequence similarity searches to detect homology relationships [3], contributing to the annotation of new genomes based on orthology inference [4], and to the identification of gene families based on the detection of paralogs, supporting hypotheses on the evolutionary mechanisms that determined their divergence [5]. Specifically, orthologs are genes in different species that started diverging from a common ancestor via evolutionary speciation [6–8]. Overall, based on the “ortholog conjecture” [9–11], or standard models of phylogenomics, genes may rapidly modify their function after duplication, leading to functionally divergent paralogs, while orthologs tend to preserve the functionality. Hence, most interest for ortholog searches is in the context of function prediction, while paralogs are commonly investigated to study gene families and function innovation. Reliable detection of orthologs in comparative genomics is also relevant to transfer annotations from characterized, reference genes, to newly sequenced genomes [10]. Paralogs instead are genes in the same species that started diverging via gene duplication [6, 8]. Gene duplication is a fundamental mechanism that determined genetic novelty in species by providing new material for gene function innovation [12, 13]. The majority of duplicated genes will vanish over time, while a smaller subset may evolve into novel or more complex functions [14].

Currently, more than 3400 eukaryotic genomes are available in public databases [15]. The availability of such a huge amount of genomic data provides relevant source of information to investigate the major mechanisms that shaped genome evolution, such as genome/gene duplication, diploidization, reshuffling, gene reduction, fusion/fission [16–19]. These studies are mainly based on sequence comparisons and aim to the detection of orthologs and paralogs from available gene annotations [8]. Consequently, the reliability of the results from these approaches is strongly related to the quality of gene and protein coding regions annotations [20]. There is a spreading interest for evolutionary analyses and for investigations on homology relationships also in plant sciences, that is proven by the dissemination of several web-based collections of orthologs and paralogs [21, 22], of public browser for the visualization of ortholog and paralog collections [23, 24], and of computational methodologies for the detection of homology relationship [25–31].

Plants evolutionary analyses revealed their propensity to evolve via large-scale duplications [32, 33], often involving polyploidization events and subsequent gene loss, with retention of paralogs [34] that favored the establishment of gene families [35]. In the model plant *Arabidopsis thaliana*, for instance, paralogs involved in signaling and transcriptional regulation mechanisms were revealed to be more often retained than other genes after the whole genome duplication events that apparently contributed in shaping the genome [35–38].

*Solanum lycopersicum* (tomato), one of the relevant crops among Solanaceae, a major family of the Asterid clade in dicotyledonous plants, is considered a model among fleshy fruit species because of its small, diploid genome, made up of 12 chromosomes. Phylogenetic studies revealed a recent genome triplication, estimated 71 (±19.4) million years ago, highlighting an interesting pattern in speciation and polyploidization in eudicot lineages [33].

*Vitis vinifera*, a perennial plant belonging to Vitaceae family (Rosids clade), is another economically important species for fruit or wine production. Recent phylogenetic studies classified the Vitaceae family as the earliest diverging lineage of Rosids [39], making it an excellent model for this clade in comparative genomics studies. The large chromosome number (19) suggested an ancestral polyploidy status of the *V. vinifera* genome [40], despite its small genome size (475 Mb) [41]. However, analyses of the grapevine genome indicated the absence of both ancient and recent duplication events in *V. vinifera* as well as in all Rosid species [32].

Asterids and Rosids approximately diverged from their last common ancestor 125 million years ago [42]. Therefore, large chromosomal rearrangements and consistent genome reorganizations should have presumably occurred. However, a comparative analysis on the genomes of *S. lycopersicum*, *Coffea canephora* and *V. vinifera* [43] revealed the presence of significant synteny fragmented into relatively small blocks of about 4 Mb between Asterids and Rosids genomes. Indeed, the highlighted synteny is particularly interesting when considering the differences in the number of chromosomes (*n)* and in the genome size of the syntenic regions (*x)* between tomato (*n* = 12, *x* = 965 Mb) and grapevine (*n* = 19, *x* = 475 Mb).

Although the interest for a suitable framework to investigate on the gene content and on the functionality of the two species, especially for what concerns fruit development and ripening, even though several years already passed since the genomes of grapevine and tomato were made accessible [32, 33], no comparative analyses for the two species gene annotations and no reference collection that could support such investigations are today available. Indeed, despite the presence of many public collections that, among several, also include tomato and grapevine sets of ortholog genes, such as PLAZA [29], Phytozome [22], Ensembl Plants [27], Inparanoid [44], EggNOG [45], GreenPhylDB [46] and OrthoDB [47], these resources are rather heterogeneous in terms of data content, since they refer to different genome annotation versions or to results from different computational approaches [48–50]. Consequently, they have still limits to favor the establishment of subsequent comparative studies that would need a reliable reference to start from.

To cover this gap and to support gene function investigations, we implemented a bioinformatics strategy (see Additional files 1, 2 and 3) to define a reliable gene comparative analysis between the two species. We integrated results from multilevel investigations of gene loci, considering gene (exons plus introns), transcript (exons) and protein sequences, available from the current most used annotations of *S. lycopersicum* (iTAG 2.4 annotation version) and *V. vinifera* (V1 annotation version). We defined a consensus set of orthologs between the two species. Paralogs for each species were also identified, again using a multilevel approach, to support gene family investigations. Networks of inter genomes and intra genome related genes were therefore defined [24, 30], together with species-specific genes from each species, which stand for genes without similarities with those from the alternative species. Functional descriptions, protein domains and association with metabolic pathways of the gene loci encoded proteins based on conventional bioinformatics similarity searches versus reference collections were exploited to reconcile the two gene annotations, to update functional information and appropriately confirm the computationally detected orthologs. The whole results were made available through a web accessible resource, ComParaLogS [51], which provides a dedicated not previously available platform to support comparative investigations of the two species gene annotations.

An overview of results achieved by the exploitation of the collection here presented integrated with gene expression data publically available for the two species reveals preliminary common and peculiar features of the two plants species genomes and provides hints on possible applications which may be supported by this resource.

## Results

### Multilevel comparison

**Fig. 1:**
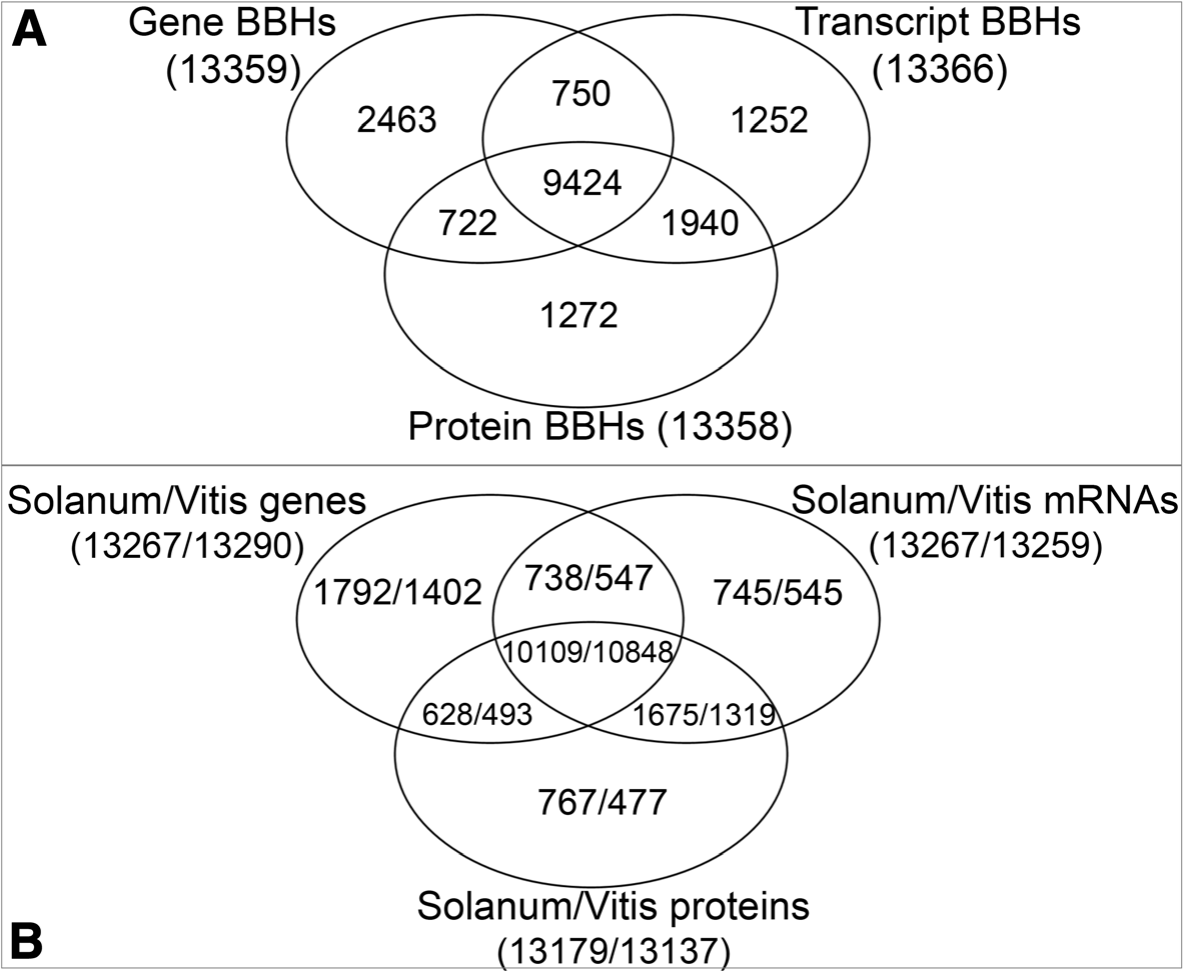
Comparison between genes, mRNAs and proteins similarity searches results. **a** Venn diagram showing differences and similarities in the number of BBHs detected using genes, mRNAs and protein sequences. **b** Venn diagram showing the number of *S. lycopersicum* genes that have an ortholog counterpart in *V. vinifera*, and vice versa

13,359 BBHs were detected using all-versus-all similarity searches on gene sequences, meaning the complete genome loci including exons plus introns, 13,366 BBHs using mRNA sequences, and 13,358 BBHs using protein sequences (Fig. 1a). We observed a consensus of 9424 relationships common to the three different levels of analyses.

**Fig. 2:**
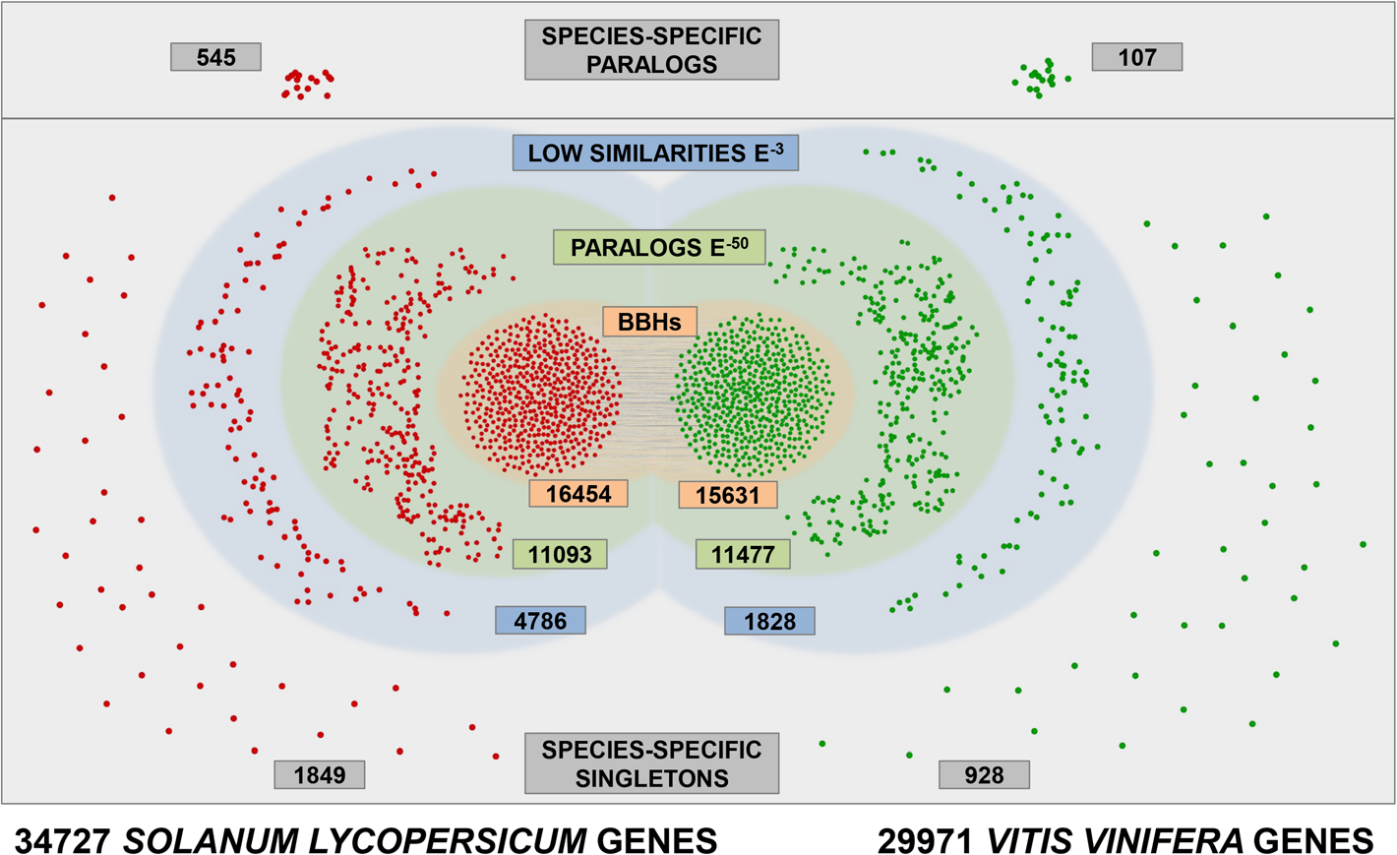
General overview of the cross comparison between *S. lycopersicum* and *V. vinifera*. Tomato and grapevine genes are represented in red and in green, respectively. BBHs are shown in orange background; paralogs detected with the stringent e-value threshold (e^− 50^) are shown in green background; low similarities detected with the loose e-value threshold (e^− 3^) are shown in blue background; species-specific genes, including paralogs and single-copy genes (singletons) are shown in light gray background

Considering the *S. lycopersicum* loci that show similarity relationships with a *V. vinifera* counterpart, and given that one locus from one species can be related to more than one locus in the other species, and vice versa, 10,109 genes are involved in all three levels of relationships, 1792 genes from tomato were involved exclusively in gene-vs-gene, 745 exclusively in transcript-vs-transcript and 767 exclusively in protein-vs-protein similarities (Fig. 1b). When considering the *V. vinifera* loci that have a relationship with a *S. lycopersicum* counterpart, we observed 10,848 genes that are involved in all three levels of relationships, 1402 genes that were involved exclusively in gene, 545 in transcript and 477 in protein similarities (Fig. 1b). Considering all levels of analyses. The results revealed that 16,454 loci of *S. lycopersicum* and 15,631 of *V. vinifera* have a BBH relationship confirmed at least by one level in the entire analysis (Fig. 2).

*S. lycopersicum* and *V. vinifera* paralogs were defined by all-against-all sequence similarity searches using genes, mRNAs and proteins. By this approach, starting from 32,085 ortholog genes (i.e. 16,454 tomato genes and 15,631 grapevine genes, respectively) we considered their associated paralogs, defined at the e-value threshold of e^− 50^, which include 11,093 Tomato and 11,477 Grapevine genes (Fig. 2). Interestingly, therefore, 8778 genes from tomato and 4899 genes from grapevine had no associated paralog at this e-value threshold.

**Fig. 3:**
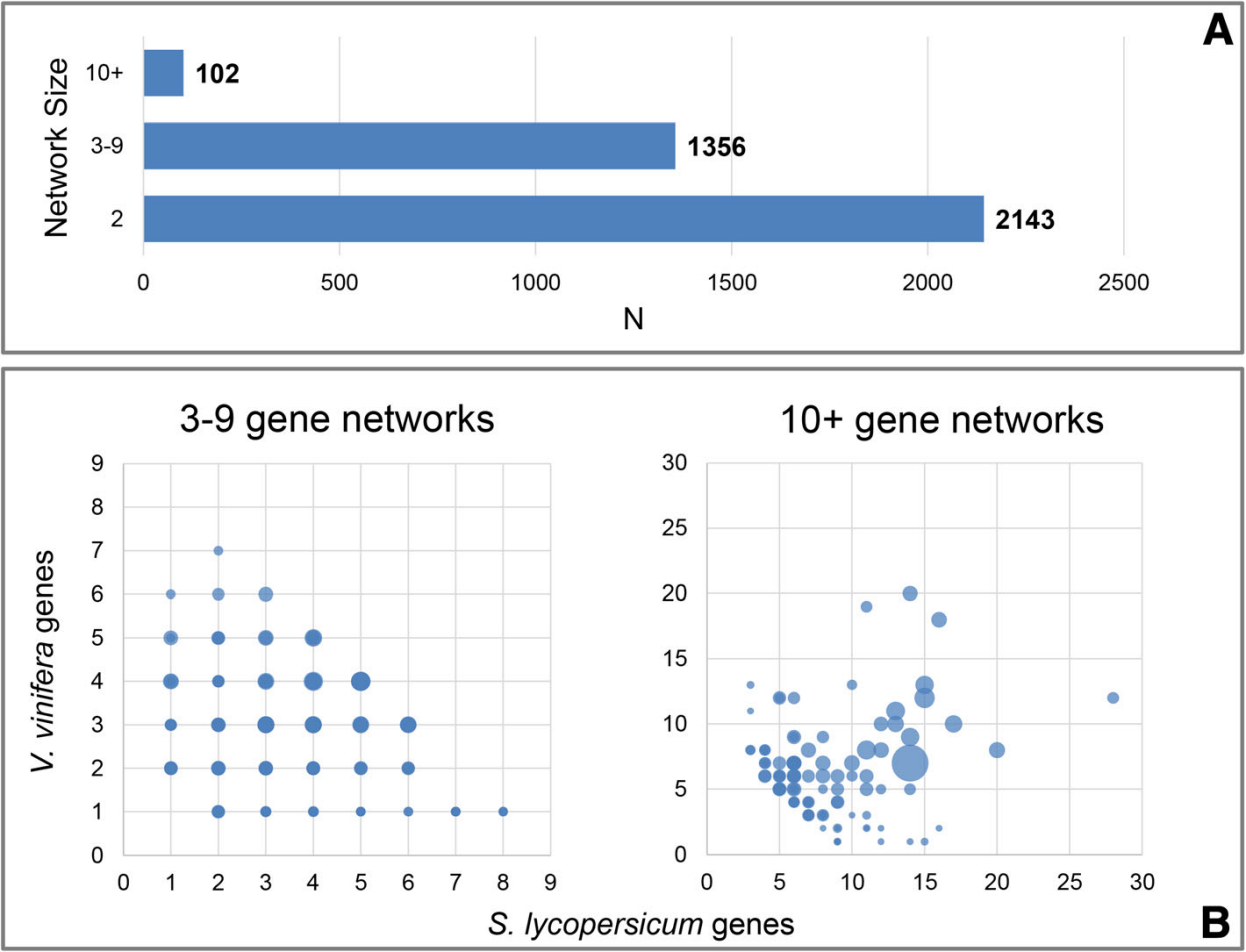
Ortholog/paralog networks detected with a stringent e-value threshold (e^− 50^). **a** Bar chart showing the number of networks classified according to their size. **b** Scatter plots showing the distribution of the networks based on the respective number of genes from *S. lycopersicum* and *V. vinifera*. The diameter of the circles is proportional to the number of BBHs inside each network

We defined 3601 distinct networks clustering orthologs and the associated paralogs from each of the two species (Fig. 3). In principle, the nodes in a network represent tomato and grapevine evolutionary related genes, or because they have a direct orthology relationship or because of a paralogy to an ortholog of the alternative species. Specifically, we defined 2143 networks made of only two genes (two-gene networks), i.e. one *S. lycopersicum* gene and one *V. vinifera* gene, both connected by one orthology relationship, representing therefore orthologs without duplication in both genomes; 1356 networks including from 3 to 9 genes, and 102 networks having a number of genes equal or higher than 10 (Fig. 3a). In Fig. 3b, an overview of the defined networks highlights the respective distribution per species based on the number of gene components from tomato and grapevine. The size of each circle in the figure shows the relative number of orthology relationships detected per network.

Looser paralogy relationships were also defined by a higher E-value threshold at e^− 3^. This settings was useful for two main reasons: although the stringent cutoff at e^− 50^ permitted the definition of the maximum number of networks [52], the possible association of genes at a looser threshold could be also of interest to investigate distantly related genes in a gene family. Moreover, the looser threshold permitted the definition of species-specific genes from the pairwise comparison as those from all the complete set of genes of *S. lycopersicum* and *V. vinifera* that had not an ortholog counterpart, neither showed similarity with an ortholog even within an e^− 3^ e-value threshold. Differences between networks detected at e-value e^− 50^ and the ones detected at e-value e^− 3^ are summarized in Table 1.

**Table 1:** Summary statistics of networks detected using different e-value thresholds

	e^−3^	e^− 50^
Total nodes	61,269	54,655
Total edges	3,699,964	1,354,314
Tomato nodes	32,333	27,547
Grapevine nodes	28,936	27,108
Orthology edges	17,823	17,823
Paralogy edges	3,682,141	1,336,491
Total networks	641	3601
Total 2-genes networks	385	2143
Total 3–9 genes networks	243	1356
Total 10+ genes networks	12	102
“Big network” nodes	59,306	43,236
“Big network” edges	3,695,231	1,328,306
“Big network” tomato nodes	31,312	21,456
“Big network” grapevine nodes	27,994	21,780

Statistics for the networks detected by using different e-value thresholds (e^−50^, for defining paralogs, and e^−3^, to define even looser similarities, respectively)

The analysis of species-specific genes in the pairwise comparison revealed 514 tomato-specific paralogs (belonging to 191 distinct networks) and 1849 tomato-specific single-copy genes (singletons), and 107 grapevine-specific paralogs (belonging to 42 distinct networks) and 928 grapevine-specific singletons (Fig. 2), which probably reflects the higher gene complexity of tomato, by a larger genome size and a larger number of estimated genes.

To further support comparative analyses, we reconciled the two gene annotations performing sequence similarity searches of the two species transcripts collections versus the Swiss-Prot protein collection, also identifying enzyme-coding genes (EC number) from each species. By this approach, within the ortholog collection here defined (Fig. 2), we assigned a protein based similarity to 13,555 out of 16,454 tomato genes and to 13,405 out of 15,631 grapevine genes.

By comparing the functional descriptions of ortholog genes we found a sufficient level of similarity in the descriptions between the orthologs, supporting the reliability of the relationships depicted by our results (Table 2). By comparing the predicted domains of tomato and grapevine proteins, the ortholog counterparts of 14,599 BBH relationships out of 17,823 (82%) share exactly all the domains, 2649 (15%) share only some domains, and 575 (3%) do not share any domain, this supporting the reliability of the relationships depicted by our results.

**Table 2:** Functional description similarity between tomato and grapevine orthologs

Functional Annotation dentity percentage	Number of orthology relationships	Description examples
100 (%)	8652	Photosystem II D2 protein Photosystem II D2 protein
80–99 (%)	1087	E3 ubiquitin-protein ligase RING1 E3 ubiquitin-protein ligase RING1-like
60–79 (%)	322	Probable pectate lyase P59 Pectate lyase
40–59 (%)	1442	Germin-like protein subfamily 1 member 14 Putative germin-like protein 2–1
20–39 (%)	4782	9-divinyl ether synthase Allene oxide synthase, chloroplastic
0–19 (%)	1629	Metal transporter Nramp6 Cinnamoyl-CoA reductase 1

Summary of the functional description similarity between tomato and grapevine orthologs, based on software inspection of matching fonts between tomato and grapevine

### Expression in the orthologs networks

A transcriptional characterization of the complete set of the BBH was also made. RNA-Seq data from three different developmental stages of fruit (see “Expression data analysis” in Methods section) were used to perform a co-expression analysis. Although tomato and grapevine rely on different mechanisms for the fruit ripening process, to perform the co-expression analysis we chose three putatively similar stages representing three critical points for fruit development and ripening in the two species. This allowed, for instance, to compare the pattern of expression from a post-setting stage to a mature stage in the two species and to highlight peculiar transcriptional behaviors.

**Fig. 4:**
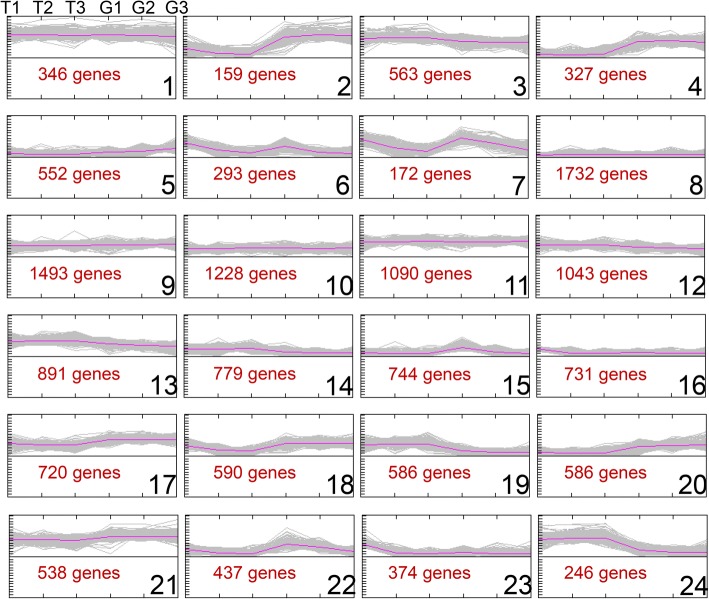
Co-expression analysis of the complete set of BBHs showing expression in at least one tissue/stage. For each of the 24 clusters identified the profiling (in grey) and the centroid (in violet) are showed. T1, T2 and T3 represent tomato fruit stages (T1 = 2 cm fruit, T2 = breaker and T3 = mature fruit) while G1, G2 and G3 represent grapevine fruit stages (G1 = post-setting, G2 = veraison and G3 = mature berry), all in physiological conditions. Numbers are used to indicate each cluster. Number of clustered genes are indicated in red

A K-means clusterization method was used and an a priori number of 24 clusters was set as input, as indicated by the Figure of Merit (FOM) [53] (see Additional file 4). The co-expression analysis (Fig. 4) highlighted that cluster 1, 3, 6, 7, 8, 9, 10, 11, 12, 13, 14, 15, 16, 21, 22 show a similar profile for the three correspondent stages of the two species. By contrast, some of the clusters highlighted an opposite pattern between the two species. For example, about 500 BBHs, from clusters 2 and 4, showed an evident grapevine preferential expression in the tested fruit stages and conversely, 800 BBHs, from clusters 19 and 24, showed a preferential expression in tomato. About 27% of the BBHs belonging to the first group (BBHs of cluster 2 and 4) and 43% of the BBHs belonging to the second group (BBHs of the cluster 19 and 24) come from the consensus dataset (homology confirmed by gene-transcript-protein levels). Details about the number of loci per cluster and the BBHs datasets included are reported in Additional file 5.

The analysis of the 24 clusters of BBHs based on the Gene Ontology enrichment highlighted that the preferentially expressed genes in grapevine fruit stages from clusters 2 and 4 are mainly related to cell wall biogenesis and organization as well as to transmembrane transporter (see Additional file 6). Further investigations on these genes could provide relevant information for understanding specificities of the maturation process of climacteric versus non-climacteric species. By contrast, no enriched GO classes were detected for preferentially expressed BBHs in tomato fruit stages from clusters 19 and 24.

In order to highlight possible differences associated with the metabolic processes of the two species, the BBHs were also mapped onto the metabolic pathways using the Mapman (v 3.6.0) software. The results from BBHs belonging to cluster 2, 4, 19 and 24 confirmed the presence of grapevine preferentially expressed genes associated with cell wall, and highlighted the presence of a group of tomato preferentially expressed BBHs associated with ethylene (see Additional file 7) and protein G.

## Discussion

In order to provide a representative cross-comparison between *S. lycopersicum* and *V. vinifera* gene loci, we considered the most used gene annotations from tomato and grapevine, i.e. iTAG 2.4 for tomato and CRIBI v1 for grapevine. These are also the most recent versions available in public comparative databases (Table 3).

**Table 3:** Tomato and grapevine genome annotation versions

Database	Tomato	Grapevine
PHYTOZOME	iTAG 2.4	Genoscope v2 = CRIBI v0
PLAZA	iTAG 2.4	Genoscope v1
ENSEMBL PLANTS	iTAG 2.4	CRIBI v1
GRAMENE	iTAG 2.4	CRIBI v1
PLANTGDB	GenBank Release 160.0	Genoscope v2 = CRIBI v0
GREENPHYL	iTAG 2.3	Genoscope v2 = CRIBI v0
EGGNOG	iTAG 2.4	CRIBI v1
INPARANOID	iTAG 2.4	CRIBI v1

Tomato and grapevine genome annotation versions available in most used comparative databases

We performed all-against-all similarity searches using the proposed multilevel approach. We updated the functional descriptions and functional domains assignments for both collections to favor comparisons, and organized all the data into a dedicated web accessible platform to support user friendly explorations of the entire collection.

**Fig. 5:**
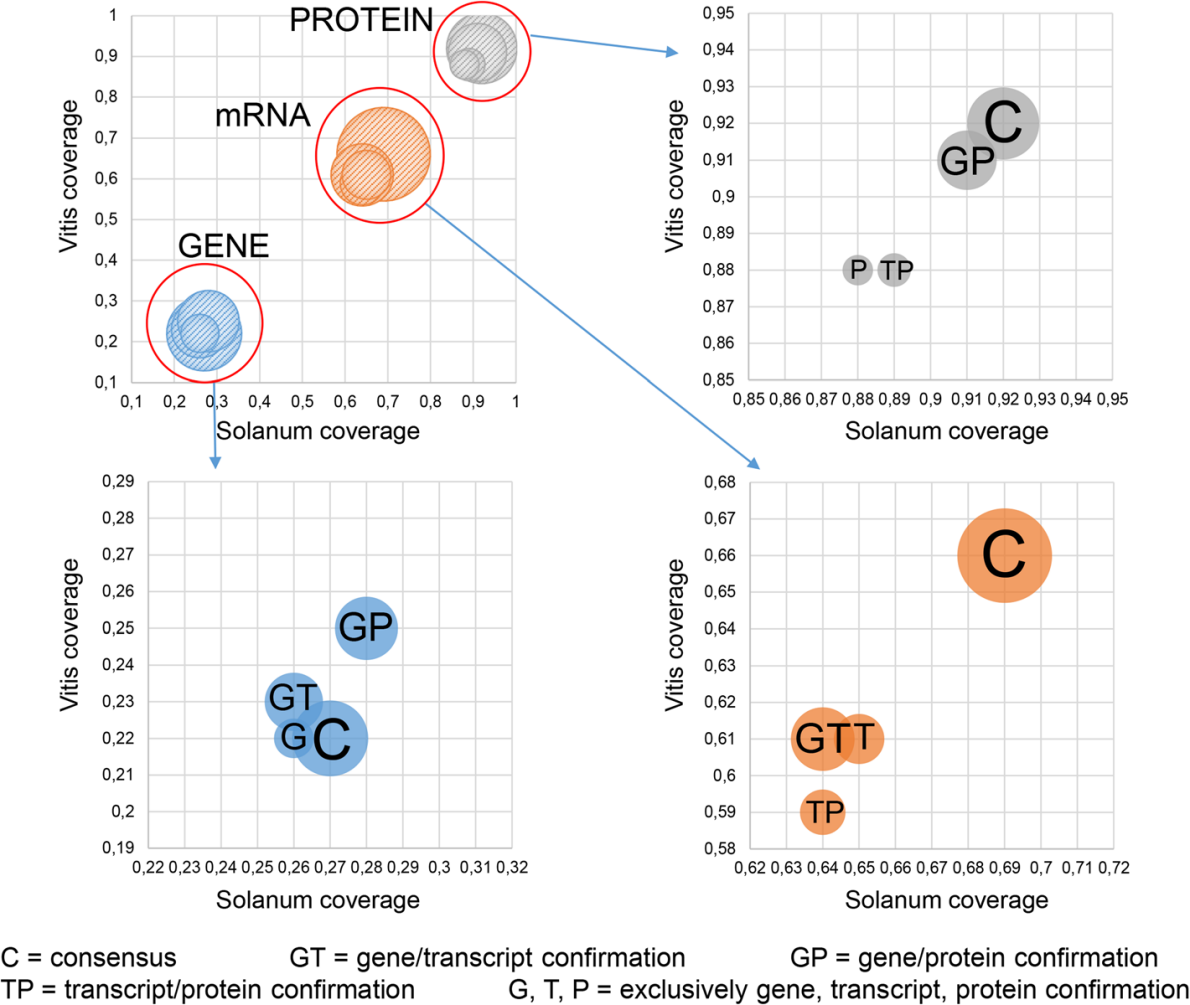
Groups of BBHs detected by each level of analysis. The confirmation level is shown: BBH detected at gene (blue), transcript (orange) and protein (grey) sequence level. The diameter of each circle is proportional to the BBH score average. The consensus groups pull together the BBHs that are common to all three different methods

The multilevel approach here proposed consisted in independent reciprocal cross-comparisons of genes, translated mRNAs and proteins. The multilevel analyses were performed setting a loose e-value threshold (e^− 3^) to determine all possible relationships. Successively, exploiting the selectivity determined by the Bidirectional Best Hits approach [54–56], genes sharing best similarity versus the counterpart, and vice versa, were defined as orthologs in each independent level of comparison. Most reliable orthologs may be considered those confirmed as BBHs by all the three independent analyses, since they represent a common consensus of BBHs defined at each level. The consensus core included 10,109 *S. lycopersicum* genes and 10,848 *V. vinifera* genes, respectively (Fig. 1b). As shown in Fig. 5, the average score of the consensus orthologs is higher than the ones for orthologs that were defined at two or at one of the levels, highlighting more dispersed similarity for BBH not included in the consensus. Indeed, it is worthy to note that due to gene, transcript or protein structure organization, the BBHs that are specific to only a single level show a lower similarity, measured by alignment scores and coverages, than the ones defined as consensus from multilevel analyses.

The definition of BBHs that have not a consensus confirmation by all levels of analyses in the two species also highlights aspects that may provide further insights on the annotated genes, providing added value information. Indeed, BBHs detected at least at one of the level of analyses reveal similarities and discrepancies which may be due to intrinsic features of the gene structure, such as divergent non protein coding regions (eg. introns or UTRs), or genome annotation limits (e.g. errors in exons or protein-coding regions predictions that could determine transcript or gene based BBHs not confirmed at protein level).

**Fig. 6:**
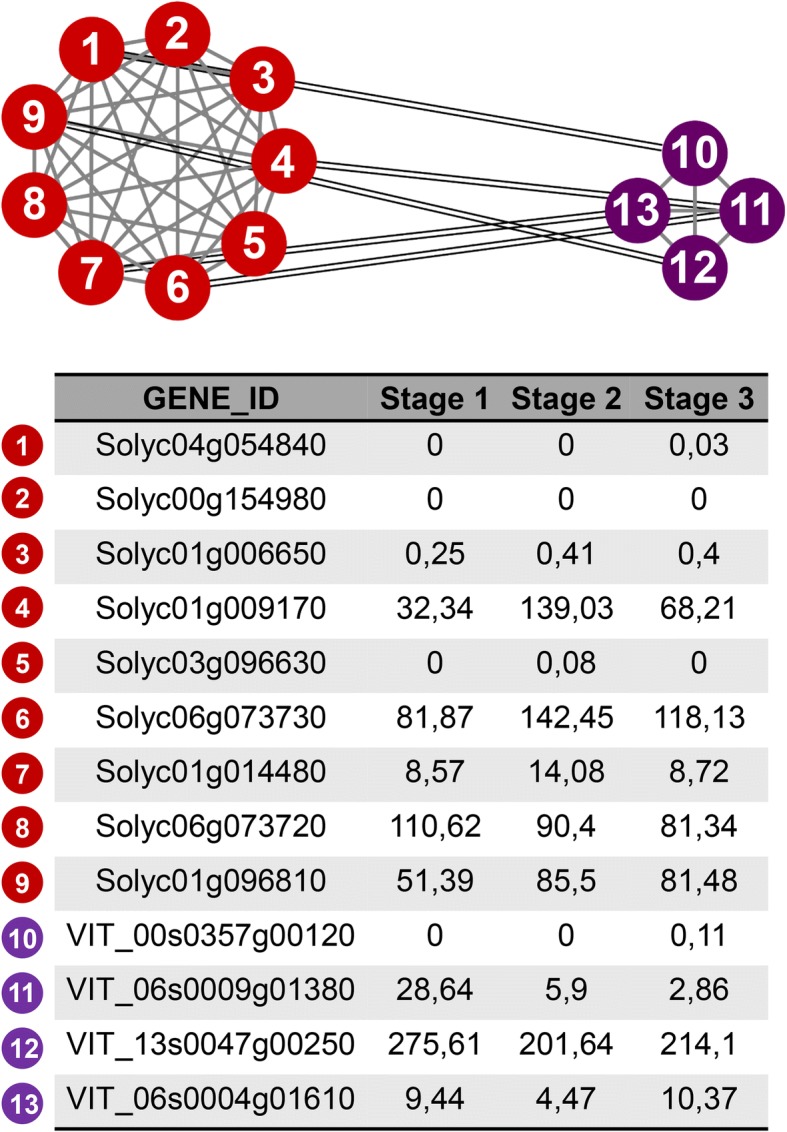
Ortholog/paralog network involving EIL transcription factor. Red circles represent tomato genes; purple circles represent grapevine genes; gray lines represent paralogy relationships; black double lines represent orthology relationships. In the table, the expression values in RPKM (Reads Per Kilobases per Million) shown for each of the considered fruit developmental stage, are associated with the genes of the network. Stage 1, 2 and 3 correspond to 2 cm fruit, breaker and mature fruit in tomato, and to post-setting, veraison and mature berry in grapevine, respectively

Paralogs, accordingly, were detected starting by independent all-against-all similarity searches of gene, mRNA and protein sequences from each species. As suggested by Rosenfeld JA and DeSalle R [52], the effects of too high e-values is grouping the majority of duplicated genes in fewer networks, while too low e-values tends to neglect many possible relationships, reducing again the number and size of the networks. Therefore, we set the e-value threshold at e^− 50^, which is discussed by Rosenfeld JA and DeSalle R [52] to be the one that maximizes the number of networks (this is also confirmed by in house testing, here not shown), since we aimed to define the maximum number of possible distinct paralog groups. This cutoff is consequently not favoring inclusive networks, i.e. association of paralog groups which contain genes with a similarity score with an estimated e-value cutoff higher than e^− 50^, and is therefore useful in this specific context, since it does not bias results aiming to reveal expansions or reductions in the number of genes in related gene families of *S. lycopersicum* and *V. vinifera*, respectively. Therefore, based on this cutoff, we selected genes similar to previously defined ortholog pairs, as well as genes associated with them by a clear similarity threshold, and grouped all of them in networks of paralogs of the two species, including direct orthologs. We summarized the general trends in Fig. 3. From the plots, the networks, that can be considered as representative of gene families that include orthologs with the species counterpart, which did not undergo significant changes in the number of genes between the two plant species are those distributed along the hypothetical principal diagonal that splits the charts. In contrast, networks that are far from this diagonal represent possible expansions or reductions in the number of genes in one of the two species compared to the counterpart (Fig. 3b). It is also possible to infer the most conserved gene families between tomato and grapevine by considering the networks including the larger number of orthologs (circles in the plots with larger diameters). The example displayed in Fig. 6 shows the expansion/reduction of a class of transcription factor, the Ethylene Insensitive Like factors, named *EIL*, involved in the ripening mechanisms. *EIL* is required to activate a cascade of processes that regulates ripening-related genes and the expansion of this family in tomato could be associated with the diverse ripening mechanisms with respect to grapevine. Six *EIL* genes were described in tomato [57] and in this survey other three genes were detected as possible paralogs (Solyc00g154980, Solyc03g096630 and Solyc04g054840). Although all three genes encode proteins with a typical *EIL-3* domain, no expression was detected in the tested physiological conditions (Fig. 6), which may suggest distinct roles among *EILs*. The distinctive expression patterns in tomato and grapevine also highlight the divergence between the two species. Only one *EIL* in grapevine (VIT_13s0047g00250) appears to have expression in fruit, with levels slightly decreasing from post-setting to mature fruit (Fig. 6). Noteworthy, for the expressed *EIL* genes this pattern is opposite to those generally found in tomato *EIL* genes, where transcripts accumulate at the onset of ripening and either decline or maintain the same level at mature stages. The tomato *EIL-1* (Solyc06g073720) shows a pattern of expression similar to that of grape VIT_13s0047g00250, undergoing slight negative regulation as fruit ripening progresses with enhanced ethylene production. Such a behavior is typical of System 1 ethylene biosynthesis, known to be responsible for basal ethylene production in fruit also in non-climacteric species [57].

We also defined intragenome similarity relationships among tomato and grapevine genes using a looser e-value threshold (e^− 3^). This was done for two main reasons. Considering a very loose e-value cutoff permitted to define all possible range of relationships between genes in a species, contributing information on possible related genes which may present lower similarity because of evolutionary divergence. Moreover, all the genes that shared no similarity with orthologs or paralogs of orthologs in the two species at a threshold of 10^− 3^ were considered species-specific genes. We detected 2394 and 1035 species-specific genes for tomato and grapevine, respectively and, among them, 545 tomato genes and 107 grapevine genes could be grouped into paralogs networks (Fig. 2), representing therefore species-specific gene families. Since 1685 tomato and 932 grapevine species-specific genes here predicted have a still unknown function, although our updating of the functional description, and 393 tomato and 84 grapevine genes are included in species-specific families, when considering a cutoff of e^− 50^, it is clear that further work to uncover their role is still needed, also at the light of understanding the function of these peculiar genes, since this was not possible by conventional bioinformatics.

In order to provide an updated and uniform description of the two gene annotations of tomato and grapevine, and to contribute to the functional annotations, we compared the two species transcripts collections with the Swiss-Prot database. Based on a blast similarity searches (see Methods section for details), we associated each transcript with the functional annotation of the most similar protein, also considering the EC number in case of similarities to enzyme. We exploited the transcripts versus protein similarity search instead of comparisons between protein sequences to overcome possible protein coding gene miss-annotation. Indeed, in association with what we previously discussed for orthologs and paralogs detections, similarity searches based exclusively on protein sequences can be limited by the lack of a correct and exhaustive definition of the protein coding regions in preliminary gene annotations from recently sequenced genomes [25, 30]. By this approach, we could associate a protein description by best similarity searches to 1763 genes that were previously defined as “unknown” in the tomato gene annotation (see Additional file 8). On the other hand, the information herein provided was essential for the gene annotation of grapevine since no reference functional description is available for this species. Moreover, the updated functional annotation, together with the subsequent association of similarity with protein domains, contributed to confirm the orthology relationships predicted in this work, since the 54% of the predicted orthologs showed similar descriptions (Table 2).

Although computationally based orthologs are rather reliable thanks to the reciprocal selective approach of a BBHs searching [48], the in-silico definition of paralogs in a species is rather controversial due to heterogeneous similarity levels between genes in specific gene families and to the impossibility to establish a unified cutoff to establish levels of evolutionary relationships. Although several more rigorous methods, and often human curated analyses, are necessary to define the belonging to a gene family, these approaches can be successfully applied to dedicate analyses of limited number of gene families and cannot be exploited for “omics level” investigations. As an example, tree-based methods can be applied to efficiently decipher orthologous and paralogous relationships, providing more detailed information, such as evolutionary distances or the order of duplication and speciation events, these methods are computationally more expensive and sometimes are outperformed by sequence similarity comparisons such as the Bidirectional Best Hit approach [6, 17, 25, 48, 50]. Alternative methodologies can provide indicative results on orthology and paralogy relationships. We compared our results to those obtained from alternative approaches implemented in some of the most referenced publicly available comparative platforms, when they included the same genome annotation version of tomato and grapevine, like Ensembl Plants [27], Gramene [31], EggNog [45] and Inparanoid [44].

The orthology and paralogy predictions in Ensemble Plants [27] and Gramene [31] rely on the same pipeline [58], based primarily on maximum likelihood phylogenetic gene trees. Although the gene tree pipeline is able to find more complex one-to-many and many-to-many relations, especially in bony fish and mammals, there is a clear correspondence with reciprocal best approaches in the simple case of unique orthologs genes [58]. Exploiting the results from these two platforms, both provide lists of paralogs. However, these two platforms do not provide information about single gene relationships, not permitting to trace the effective link among the different genes in the list. Concerning orthology relationships, instead, Ensemble Plants and Gramene provide lists of pairwise orthologs that we could compared to our results in more details. The orthologs detected by Ensembl Plants/Gramene pipeline in Tomato and Grapevine (31798) are more than those from our approach (17823) (see Additional file 9). This discrepancy relies on the fact that in the first group the consideration of orthology relationships is not restricted to unique orthologs as the ones defined by the best reciprocal hit approach, but includes also other matches that define one to many and many to many relationships. Among the orthologs that are in common (14858) we found almost all the relationships originally included in our consensus group of relationships (8996 among 9424, Fig. 1 and Additional file 9). However, there is a group of “consensus” relationships (428) that was not detected by the Ensembl Plants/Gramene pipeline. We report in Table 4 some examples from this group of 428 “consensus” relationships showing the BLAST score and e-value, highlighting sequence similarity for the orthologs at each of the level of analysis here performed, comparing the orthologs we detected with those from the other platforms here considered. The only relationships inferred by Ensembl Plants/Gramene that included at least one of the two orthologs showed in the examples (Tab. 4A-B) have different similarity levels, having the compared sequences different lengths, and resulting in alignments with different coverages and identity percentages (Tab. 4). Moreover, the third example (Tab. 4C) shows a relationships inferred by our approach only at gene and transcript level, not at protein level. The need of not neglecting such relationships in the list of orthologs is confirmed also by the high similarity level in terms of both score and e-value here detected, confirming the usefulness of a multilevel approach when single protein based comparisons fail to detect relevant similarities hiding relationships at genome level.

**Table 4:** Statistics of alignments based on sequence similarity of gene, transcript and protein comparisons

(A) EXAMPLE 1								
	Query length	Subject length	Query coverage	Subject coverage	Identity/alig. Length	Positives/alig. Length	Score	e-value
Solyc07g008880.2 versus VIT_09s0002g07070								
Gene vs Gene	12,772	12,589	7556/12772	7575/12589	6338/7556	–	6808	0
mRNA vs mRNA	2559	2476	2289/2559	2289/2476	2152/2289	2200/2289	5385	0
Protein vs Protein	2384	2348	2336/2384	2340/2348	2267/2343	2306/2343	4737	0
Solyc06g043160.1 versus VIT_09s0002g07070								
Gene vs Gene	159	12,589	154/159	154/12589	118/154	–	82	3e^−15^
mRNA vs mRNA	53	2476	53/53	53/2476	43/53	47/53	111	7e^−26^
Protein vs Protein	52	2348	52/52	52/2348	42/52	46/52	95	5e^−24^
(B) EXAMPLE 2								
Solyc01g111530.2 versus VIT_03s0038g02340								
Gene vs Gene	11,273	26,647	5531/11273	5540/26647	4318/5531	–	3116	0
mRNA vs mRNA	2044	1986	1681/2044	1681/1986	1287/1681	1447/1681	2927	0
Protein vs Protein	1860	1897	1860/1860	1896/1897	1500/1914	1669/1914	2689	0
Solyc01g111530.2 versus VIT_04s0023g03830								
Gene vs Gene	11,273	10,303	3225/11273	3228/10303	2548/3225	–	2024	2e^−98^
mRNA vs mRNA	2044	1932	1499/2044	1499/1932	1093/1499	1258/1499	2558	0
Protein vs Protein	1860	1811	1764/1860	1799/1811	1190/1830	1407/1830	2210	0
(C) EXAMPLE 3								
Solyc01g007530.2 versus VIT_10s0092g00760								
Gene vs Gene	1813	3870	1024/1813	1034/3870	910/1024	–	1127	0
mRNA vs mRNA	353	318	160/353	160/318	130/160	141/160	336	4e^−54^
Protein vs Protein	SEQUENCE SIMILARITY NOT DETECTED							

The first three alignments of each example lead to the prediction of an orthology relationship by the multilevel approach proposed in this work: (A) Solyc07g008880.2 versus VIT_09s0002g07070, (B) Solyc01g111530.2 versus VIT_03s0038g02340 and (C) Solyc01g007530.2 versus VIT_10s0092g00760. The second triplets of alignments of (A) and (B) lead to the prediction of an orthology relationship by The Ensembl Plants / Gramene pipelines involving the same tomato or grapevine gene implicated in the relationship inferred by our approach (Solyc06g043160.1 versus VIT_09s0002g07070 and Solyc01g111530.2 versus VIT_04s0023g03830). Query length and query coverage are referred to the tomato gene loci, subject length and subject coverage are referred to grapevine gene loci

Homology comparisons in EggNog [45] are based on the Smith-Waterman algorithm [59], combined with composition-based score adjustment such as in BLAST [21]. After grouping in-paralogous proteins, orthology between proteins are assigned by joining triangles of reciprocal best hits initially involving three different species [45]. Unfortunately, the EggNog database provides lists of multi-species clusters, making a gene by gene comparison of the orthologs collections impossible.

Orthology groups in Inparanoid [44] are defined using the pairwise similarity scores calculated using the NCBI-BLAST [21] between two complete proteomes. An orthology group is initially composed of two so-called seed orthologs that are found by Bidirectional Best Hits between two proteomes. More sequences are added to the group if there are sequences in the two proteomes that are closer to the corresponding seed ortholog, than to any sequence in the alternative proteome. These members of an orthology group are called inparalogs [44]. Also the Inparanoid database, however, provides lists of group of orthologs, making a gene by gene comparison of the orthologs collections impossible.

**Fig. 7:**
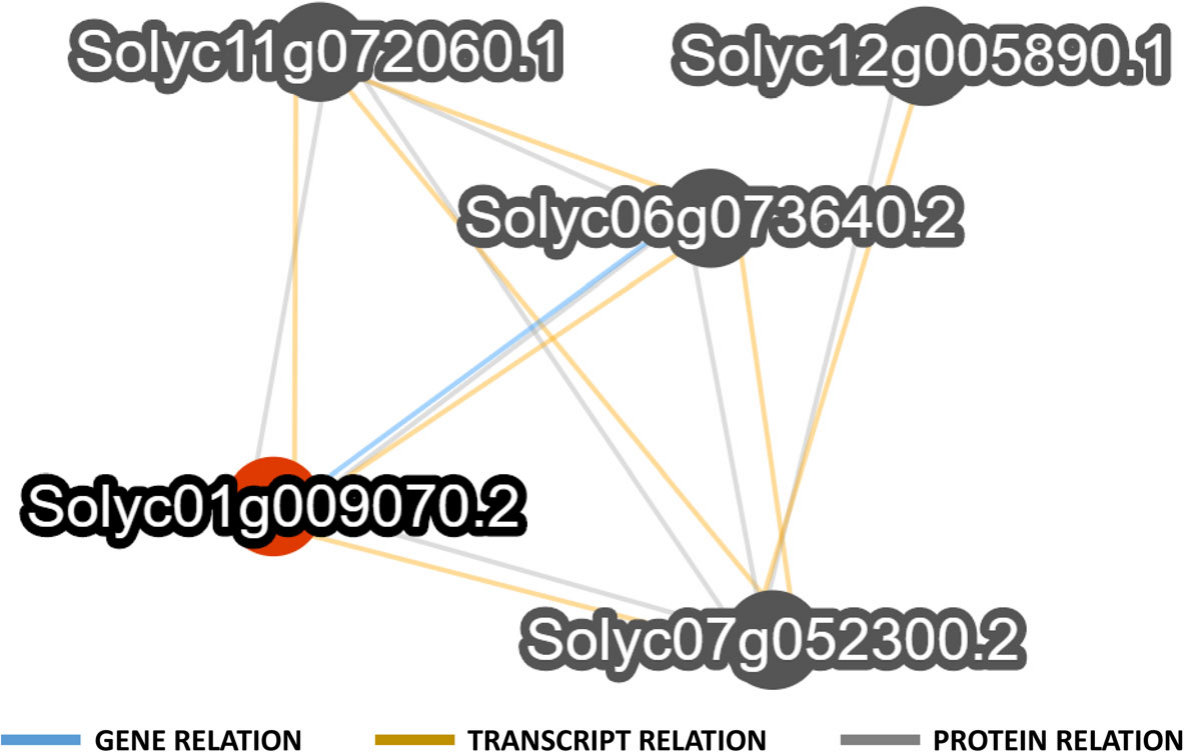
Example of a network showing different type of relationships. Network extracted from our database (http://biosrv.cab.unina.it/comparalogs/gene/search) showing a group of 5 GAMYB transcription factors sharing different type of relationships. Gene relationships are shown in blue; transcript relationships are shown in dark yellow; protein relationships are shown in grey

A substantial difference between all the presented pipelines and our approach is their exclusive use of protein sequences to infer the orthology relationships. Our multilevel analysis, instead, exploits all the levels of a gene information content detecting a consensus and a comprehensive approach to get a more reliable overview of gene loci similarities especially in still preliminary gene annotations. As an example, 4465 of our partially confirmed sequence similarities based on transcript and/or genes do not reveal a sequence similarity when the encoded proteins are compared. Moreover, in our analysis, the definition of networks of related genes allows to investigate not only which are the associated genes, but also the level at which the relationships were confirmed (Fig. 7). None of the four considered resources, finally, allows the immediate identification of species-specific genes for each species or presents reconciled functional descriptions on the same protein database reference to straightforward compare genes and their annotations.

Since orthologs are expected to share the same functionality in the species in which they are detected, this should be also confirmed, presumably, by a similar expression patterns. Dissecting the expression patterns as a way to track gene functionality after the speciation event [60] revealed that more than the 70% of BBHs identified in this study between tomato and grapevine showed similar expression patterns. Interestingly, however, some of the clusters also highlighted an opposite pattern between the two species. The transcriptional differences detected could provide some hints to further investigate the evolving mechanisms underlying gene regulation in the two species characterized by two distinct ripening mechanisms. The performed Gene Ontology enrichment showed that two clusters of grapevine preferentially expressed BBHs, contained genes related to cell wall biogenesis and organization as well as to transmembrane transporter (see Additional file 6). Cell wall modification is a relevant phase of ripening, both in climacteric and non-climacteric species, although softening of the fruit is particularly pronounced in climacteric species [61]. Genes encoding polygalacturonases (CmPG), glucan endo-1,3-β-glucosidases (CmGLU) and β-d-xylosidases (CmXYL) are known to be induced by ethylene and several of these genes were more strongly up-regulated during ripening in climacteric than in non-climacteric species. Increased expression and activity of these enzymes is expected to promote cell wall degradation and softening of the fruit. However, recent studies on *Cucumis melo* [61] showed that other cell wall related genes (e.g. a fascilin-like arabinogalactanan protein gene) were more highly expressed in non-climacteric than in climacteric species, suggesting that loss of fruit firmness in non-climacteric species is mediated by a different set of enzymes and is probably independent of ethylene signaling. Here, a group of 12 expansin genes was found to be preferentially expressed in grapevine and could represent a set of interesting genes for studying non-climateric processes related to ripening-associated cell wall modification. The subsequent mapping of the BBHs on the metabolic pathways confirmed the presence of grapevine preferentially expressed genes associated with cell wall, and showed the presence of a group of tomato preferentially expressed genes associated with ethylene (see Additional file 7) and protein G. This supports the idea that evolutionary changes affected the pattern of expression, presumably differentiating some specific processes, although preserving a sequence similarity, allowing in fact to still infer an orthology relationships. Divergence in gene regulation, rather than on the protein encoding loci, should be also investigated as one relevant driving force affecting species differentiation [62–64].

We organized a public database which contains all the presented data, available at [51]. The data are also cross-linked to reference public genome resources of the two species. A network-based description of gene relationships integrated with expression data here defined provides a preliminary effort towards integrated omics data, paving the way to the depiction of multilevel information, for a comparative “regulomics” approach [65].

## Conclusion

This work aimed to define a dedicated framework to support comparative analyses between Tomato and Grapevine gene annotations. It also presents a suitable approach to deeply analyze relationships between two species gene loci and to detect orthologs, paralogs and species specific genes between the two species, organizing the related genes into gene networks.

The approach is based on a multilevel comparison of gene, transcripts and protein sequences to define a robust and reliable collection, even in presence of preliminary annotations. The functional annotation, based on protein similarities and protein domains prediction, is also proposed as fundamental to reconcile the data and favor functional description comparisons.

To support user friendly exploration of the data, we implemented a dedicated web based platform. To our knowledge, no similar inclusive results are available elsewhere.

Although tomato and grapevine are phylogenetically distant species, we showed the presence of an evident core of reliable ortholog genes representing the 47% of the gene content of tomato and 52% of grapevine, respectively. Interestingly, more than 70% of these genes also showed similar fruit expression patterns, confirming a similar mechanism of gene transcriptional response/activation in these two distant species. However, transcriptional differences in some patterns were also highlighted. This provides relevant hints to investigate on the mechanisms underlying interesting traits, including the processes associated with fruit development and ripening.

We also defined that 6,9% of tomato genes and 3,5% of grapevine genes resulted as species-specific in the cross-comparison, highlighting the presence of specific gene families too, which expanded after the speciation events. Since all the species-specific genes resulting from the cross-comparison appear to be with a still unknown function, more detailed functional studies will clarify aspects on their role within the respective genomes.

Thanks to this effort, we also assigned a function to the 25% of previously unknown tomato genes (1763 of 7124), improving the iTAG functional annotation. On the other hand, grapevine genes were enriched with an updated similarity based functional description, providing a useful information lacking for the interested community.

Beyond providing a reference framework for comparative analysis of two relevant fleshy fruit crop species, our effort also depicts a powerful bioinformatics approach. Such an integrated framework, accompanied by a multilevel approach to detect similar loci, in fact, aims to overcome the limits of preliminary gene annotations and techniques exclusively based on protein sequences. This is fundamental in comparative studies of species with draft gene annotations, which are nowadays dramatically spreading thanks to the fast evolving field of genome sequencing, or for comparisons of genes which experienced drastic evolutionary changes in the protein coding regions of similar loci.

## Methods

### Data sets

Gene, transcript and protein collections for *S. lycopersicum* [33] (release iTAG 2.4) and *V. vinifera* [66] (release CRIBI V1) were downloaded from the Solanaceae Genomics Network website [67] and from the CRIBI website [68], respectively.

Predefined Orthology relationships for tomato and grapevine were downloaded from Ensembl Plants (Release 39) [27], Gramene (build 57) [31], EggNog (v4.5) [45] and Inparanoid (v8) [44].

### Orthology and paralogy prediction

All-against-all sequence similarity searches between *S. lycopersicum* and *V. vinifera* genes, mRNAs and proteins collections were independently performed using the appropriate BLAST analysis (BLASTn, tBLASTx and BLASTp, respectively) (v2.2.29+) [21]. With the aim of including the widest significant subset of relationships, all the similarity searches were carried out setting an expect-value (e-value) cut-off at e^− 3^. In order to identify orthologs based on each analysis*,* we implemented a dedicated Python (v3.3.3) based software evolving from *Transcriptologs* [25], a transcriptome-based approach to predict orthology relationships. The software takes as input the output of the different BLAST based similarity searches, accordingly to what described in Additional file 1. It implements a Bidirectional Best Hit (BBH) approach [54–56], which establishes that gene x_i_ and y_i_, from species X and Y, respectively, are the best putative orthologs if x_i_ is the best hit of y_i_, and y_i_ is the best hit of x_i_ in an all-against-all similarity searches [69]. By this approach, in some cases one gene of a species may have relationships with more than one gene of the compared species, because the x_i_ gene may find more genes with the same alignment score, and these genes finds x_i_ as the Best Bidirectional Hits in the opposite search. To not arbitrarily exclude relationships when more than one alignments occurred with the same best score, although the orthology detection approach was designed to detect only one ortholog for each compared gene, we included the occurred multiple relationships to the list of detected orthologs.

All the similarity searches were carried out using two different settings of the e-value cut-off. The more stringent e-value at e^− 50^ was used to define paralogy relationships, as described in Additional file 2, based on a study designed to maximizes the number of predicted gene families [52], and the less stringent e-value at e^− 3^ was used to detect sequences that could share even a loose similarity with orthologs of the other species. The genes not included even in this last threshold after the cross comparison were considered to be species-specific genes.

### Networks construction and species-specific gene collections

Networks of genes based on their similarity relationships were built. The network construction process relies on the BBHs and on the putative paralog genes detected with a stringent e-value threshold of e^− 50^, defining related gene-families between the two species. This procedure organizes all the groups of connected components into distinct graphs by using the NetworkX package (v1.9) (see Additional file 3) [70]. Each node represents a gene and each edge represents an orthology or paralogy relationship based on gene and/or mRNA and/or protein similarity. Cytoscape software (v3.4) [71] was used to show the networks organization.

The tomato-specific and grapevine-specific genes were defined filtering out all the genes, mRNAs and proteins that share at least one orthology relationship with the counterpart, or a paralogy relationship with an ortholog. In this specific case, even the looser threshold for paralog detection at the e-value of e^− 3^ was considered, to exclude any level of possible similarity of tomato and grapevine genes with the remaining ones.

### Protein similarity assessment

To set up an useful comparative framework based on updated and common descriptions of gene functions, a sequence similarity search versus the Swiss-Prot sequence database of the complete mRNA collections of *S. lycopersicum* and *V. vinifera* was performed using the tBLASTn program [21], setting an e-value cut-off at e^− 3^ and the “max_target_seqs” parameter to 500. Only the alignments with a minimum of 90% identities and 90% coverage were retained for subsequent analyses. Among the accepted results, the tomato or grapevine mRNAs that matched a Swiss-Prot protein associated with an Enzyme Commission number (EC number) were also identified.

In order to confirm the orthology relationships predicted by a BBH approach, we implemented a dedicated Python (v3.3.3) software to compare and match the strings of the updated functional descriptions of each ortholog couple.

An InterProScan (version 5.28–67.0) analysis [72] was performed on the entire protein sequence collection of both tomato and grapevine (activating the “iprlookup” parameter). This software allows amino acid sequences to be scanned against the InterPro database [73], a reference collection for protein domains.

### Expression data analysis

Processed RNA-Seq libraries (Accession SRP010775) from tomato were downloaded from the NexGenEx-Tom database [74]. The grapevine RNA-Seq collections (Accession SRA009962) from Zenoni et al. [75] were preprocessed with the same methodology as in [74]. The two collections include three stages of *S. lycopersicum* cv. Heinz (2 cm fruit, breaker and mature fruit) and grapevine *V. vinifera* cv. Corvina (post-setting, veraison, mature berry) fruit in physiological conditions. The gene expression levels were defined based on the mRNA loci both for tomato [76] and grapevine (12X v1) annotations (this last by CRIBI [68]).

Read counts were normalized by Reads Per Kilobases per Million (RPKM) for each tissue/stage. Genes with expression level below 1 RPKM were considered as not expressed. Due to the differences in ranges of expression, and in order to have a better visualization of the profiling, the log2 transformation was applied for cluster analyses.

The identification of the expression profiles was performed by the K-means clustering method [77]. The Pearson correlation coefficient for pairs of gene expression profiles was used as the distance metric. The Figure of Merit (FOM) plot [53] was used to define the most appropriate number of clusters to be used for the K-means clustering. For each cluster a Hierarchical sub-clustering (HCL) [78] was performed using the Euclidean distance on the basis of expression levels. Both the clustering methods are implemented in the MultiExperiment Viewer (MeV) software [79].

A Gene Ontology enrichment analysis of the BBH subset was conducted using the online software AGRIGO 1.2 [80, 81]. A hyper-geometric test was used to compare the input test versus the reference list and a Hochberg multi-test adjustment method with a significance level of 0.05 was applied. In addition, the MAPMAN software (v 3.6.0) [82] was used for displaying expression data sets onto diagrams of metabolic pathways and processes.

## Additional files

Additional file 1: Pseudocode of the Orthologs search. The analysis implements the search for Bidirectional Best Hits considering as input the results of two BLAST based analyses. (ZIP 968 kb)

Additional file 2: Pseudocode of the Paralogs search. The analysis implements the identification of paralogs of a given species at a given e-value threshold (k) considering as input the results of a BLAST based analysis. (ZIP 1625 kb)

Additional file 3: Pseudocode of the Networks definition. The analysis implements the definition of networks by the NetworkX package (v1.9) considering as input files the results of Orthologs and Paralogs searches. (ZIP 6164 kb)

Additional file 4: Figure of Merit. The graph represents the measure of fit of the expression patterns for the clusters produced. The horizontal red line represents the threshold used to detect the best number of clusters. (ZIP 974 kb)

Additional file 5: Details about BBHs and confirmation levels for each specific cluster detected by our expression analysis. Gene content per cluster and details of the BBHs which are included in each class according to the levels of similarity that confirmed their orthology relationships. (XLS 51 kb)

Additional file 6: Hierarchical graph related to molecular function and biological process of enrichment analysis of the clusters 2 and 4. The graph contains statistically significant terms. The nodes in the image are classified into ten levels, which are associated with corresponding specific colors. The smaller the term adjusted *p*-value, the more significant statistically, with nodes with darker and redder color colors. Inside the box of the significant terms, the information includes: GO term, adjusted p-value, GO description, number of items mapping the GO in the query list and background, and total number of query list and background. (TIF 7463 kb)

Additional file 7: Mapping of the BBHs on the metabolic pathways. Mapping of the BBHs belonging to the cluster 2, 4, 19 and 24 on the metabolic (A) and regulation (B) pathways by using the MAPMAN software. Each square represents a gene preferentially expressed in grapevine (blue) or in tomato (red). (TIF 9221 kb)

Additional file 8: Protein descriptions of tomato “unknown” genes. List of unknown genes in the iTAG annotation, enriched with protein functions and descriptions assigned by similarity to UniProt database. (XLS 306 kb)

Additional file 9: Comparison between orthology relationships predicted by EnsemblPlants / Gramene platforms and by our multilevel approach. Among the orthology relationships predicted by the multilevel approach, the use of gene, transcript and/or protein sequences for each gene locus is also indicated. The contemporary use of gene, transcript and protein sequences for each gene locus is indicated as “consensus” in the figure. (TIF 1254 kb)

## Abbreviations

BBH: Bidirectional Best Hit
EIL: Ethylene Insensitive Like
GO: Gene Ontology
