# Supplementary figures and images for: Multilevel comparative bioinformatics to investigate evolutionary relationships and specificities in gene annotations: an example for tomato and grapevine
Source: BMC Bioinformatics. 2018 Nov 30;19(Suppl 15):435. doi: 10.1186/s12859-018-2420-y (PMC6266932; doi:10.1186/s12859-018-2420-y)

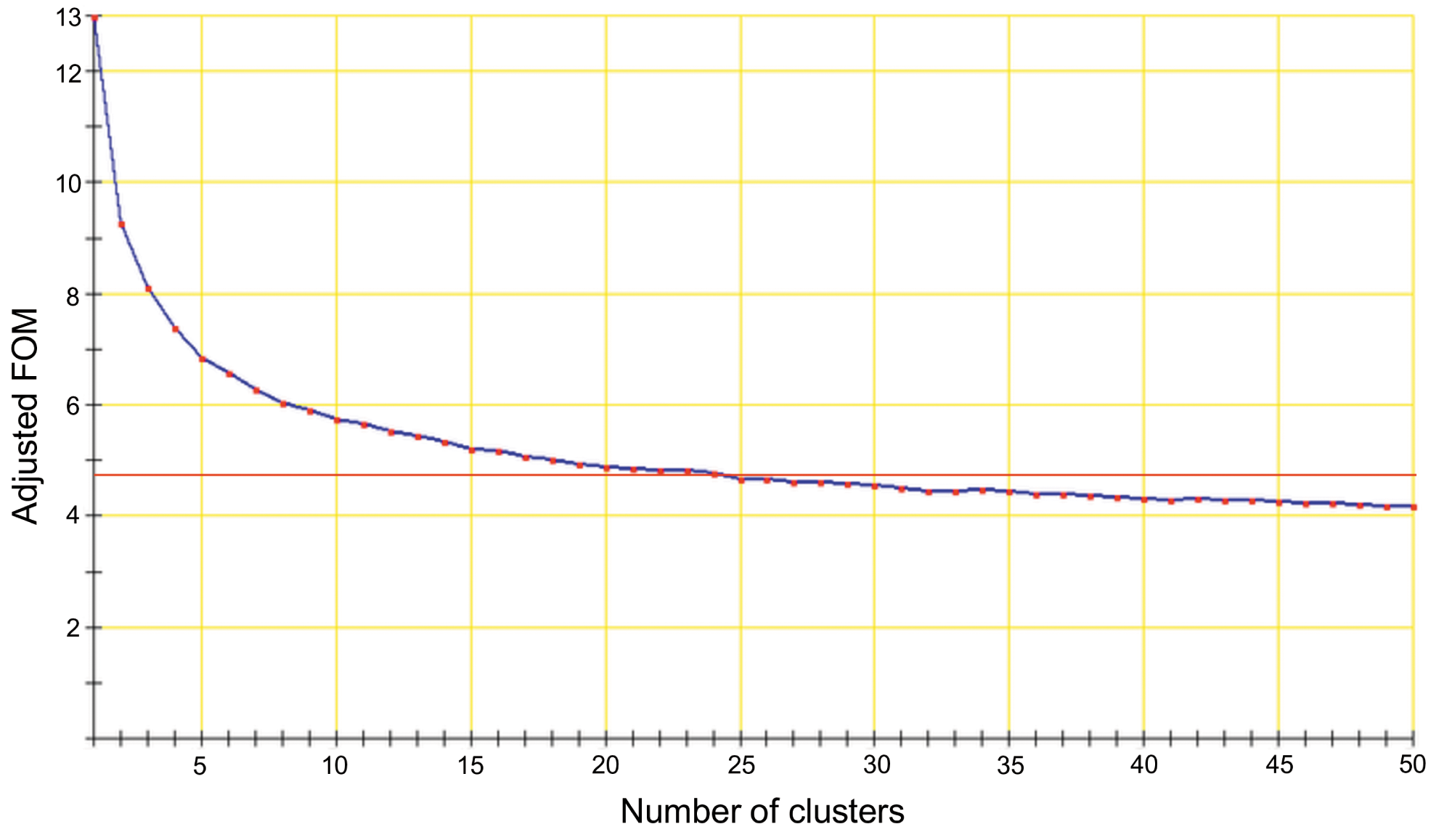

Supplement: Supplementary file 1 — Pseudocode of the Orthologs search. The analysis implements the search for Bidirectional Best Hits considering as input the results of two BLAST based analyses. (ZIP 968 kb) [file 12859_2018_2420_MOESM1_ESM.zip › S8-S1.tif]

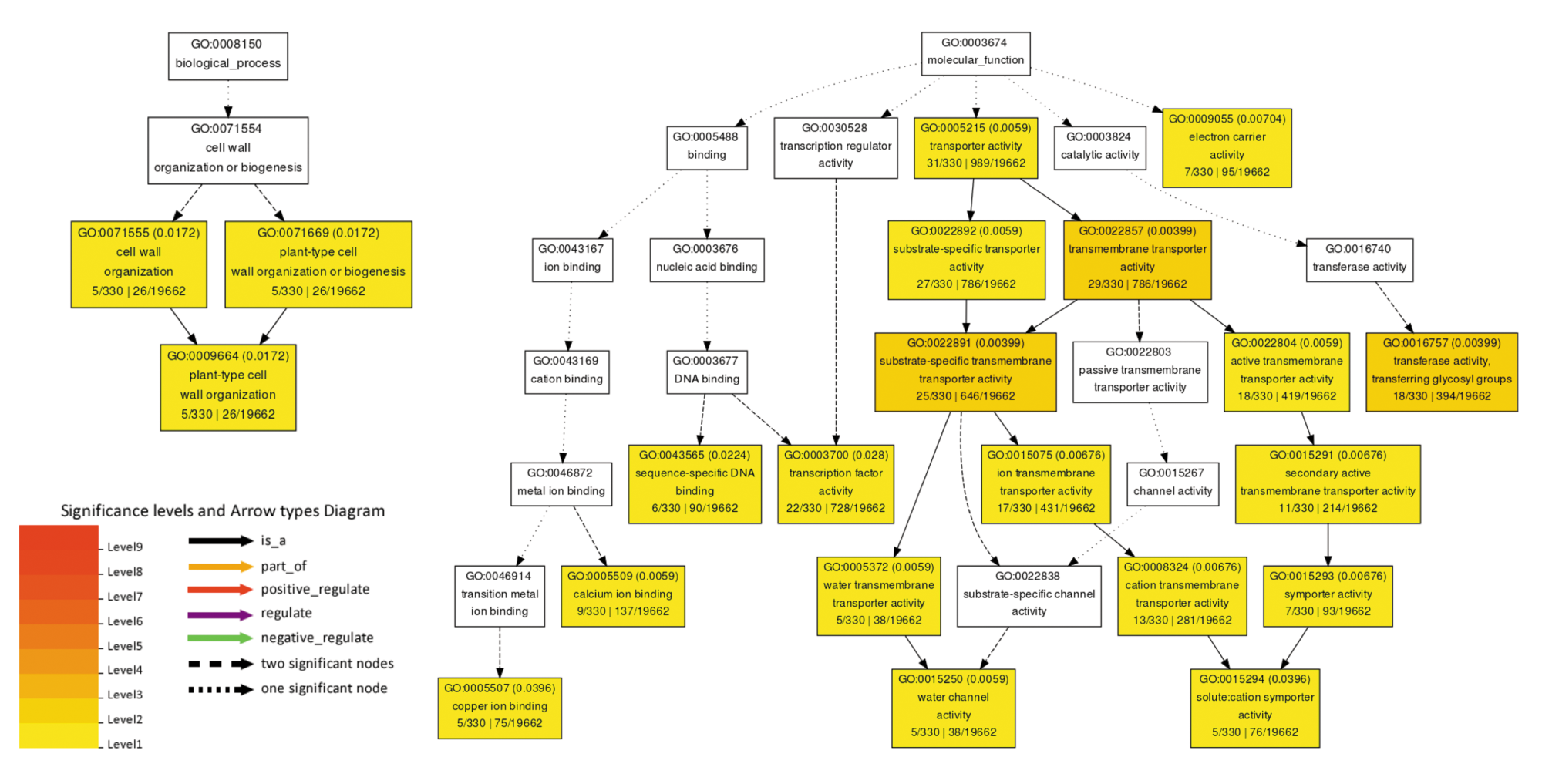

Supplement: Supplementary file 2 — Pseudocode of the Paralogs search. The analysis implements the identification of paralogs of a given species at a given e-value threshold (k) considering as input the results of a BLAST based analysis. (ZIP 1625 kb) [file 12859_2018_2420_MOESM2_ESM.zip › S8-S2.tif]

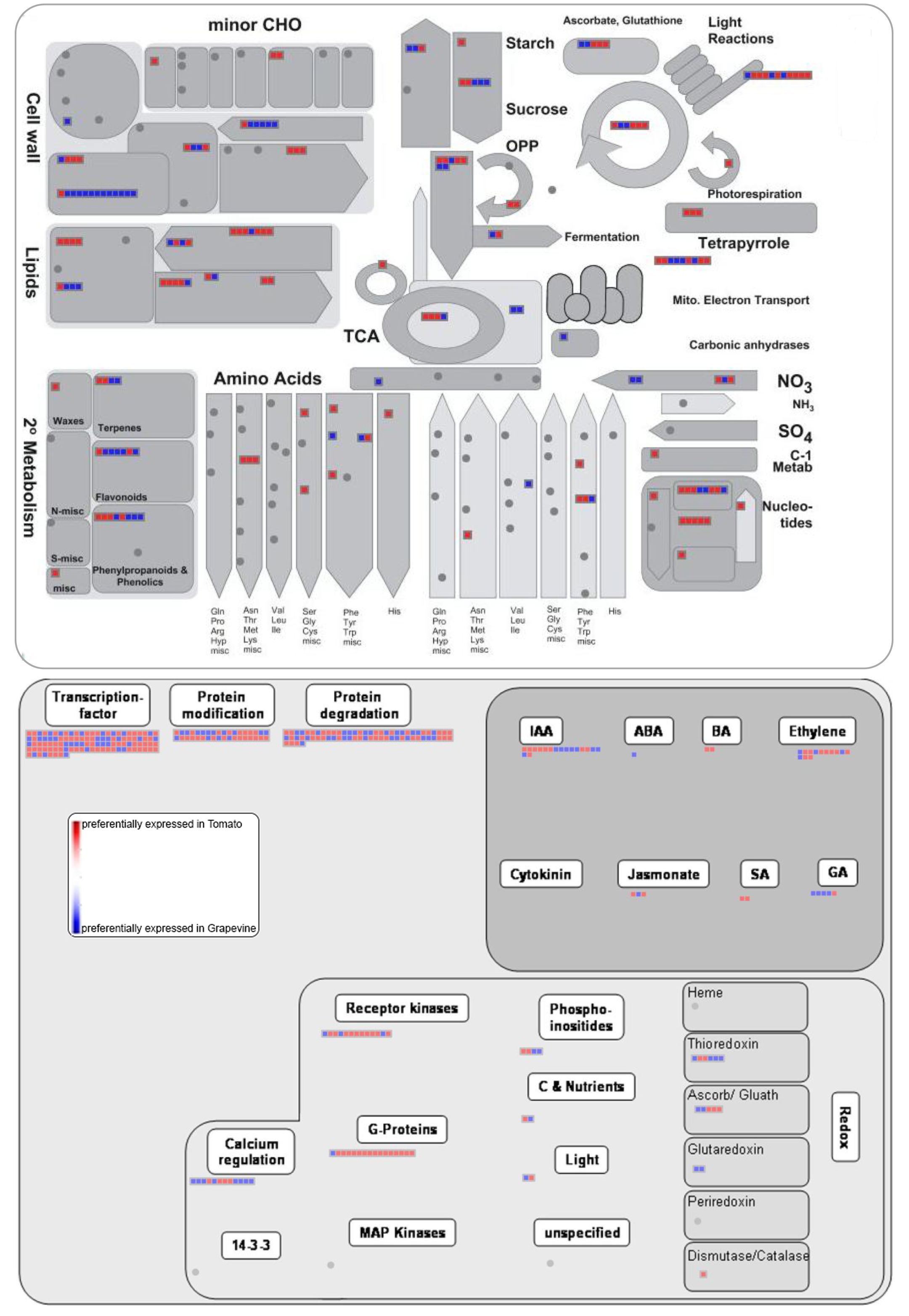

Supplement: Supplementary file 3 — Pseudocode of the Networks definition. The analysis implements the definition of networks by the NetworkX package (v1.9) considering as input files the results of Orthologs and Paralogs searches. (ZIP 6164 kb) [file 12859_2018_2420_MOESM3_ESM.zip › S8-S3.tif]

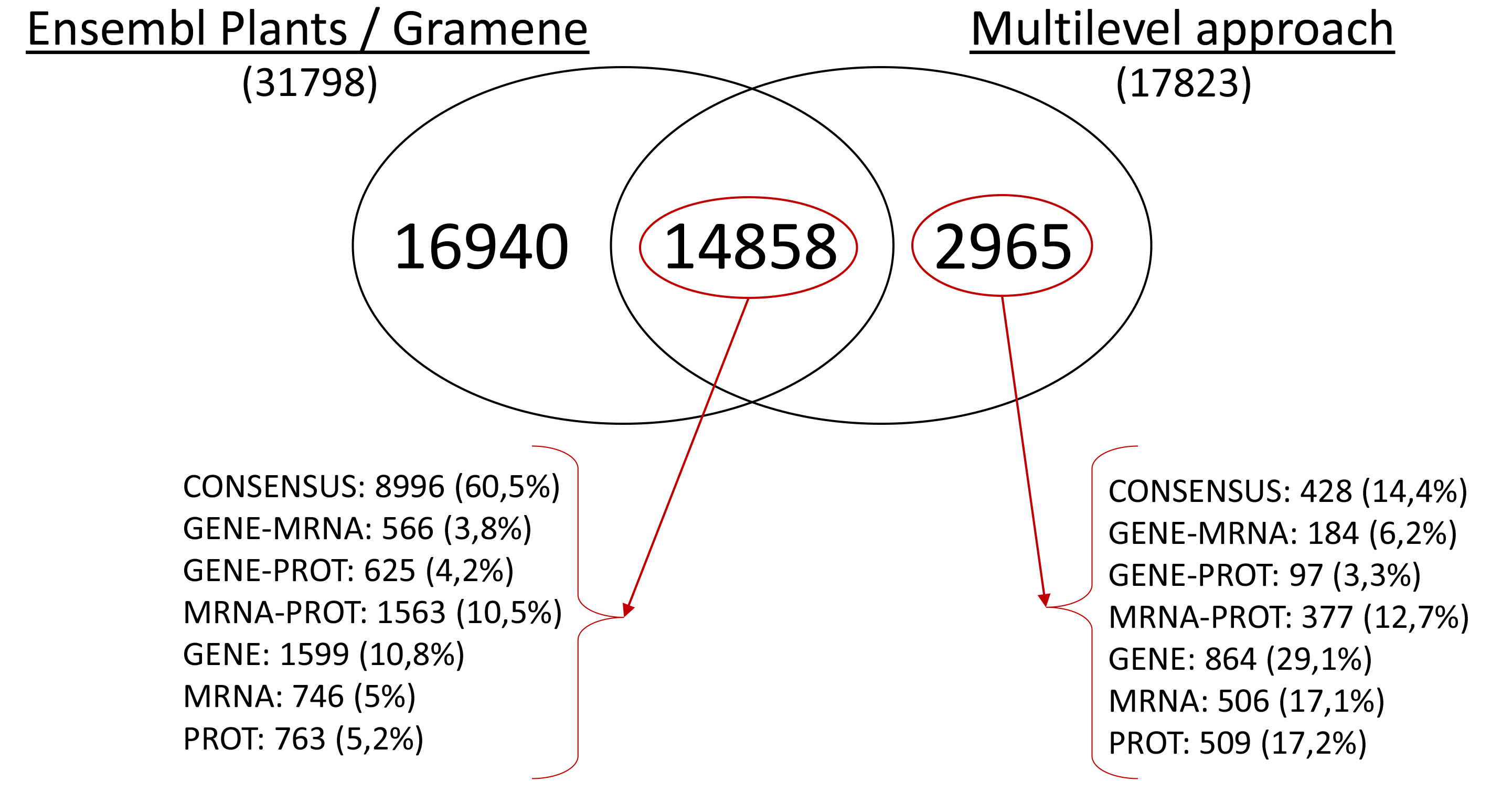

Supplement: Supplementary file 9 — Comparison between orthology relationships predicted by EnsemblPlants / Gramene platforms and by our multilevel approach. Among the orthology relationships predicted by the multilevel approach, the use of gene, transcript and/or protein sequences for each gene locus is also indicated. The contemporary use of gene, transcript and protein sequences for each gene locus is indicated as “consensus” in the figure. (TIF 1254 kb) [file 12859_2018_2420_MOESM9_ESM.tif]
