# Supplementary material for: Multilevel comparative bioinformatics to investigate evolutionary relationships and specificities in gene annotations: an example for tomato and grapevine
Source: BMC Bioinformatics. 2018 Nov 30;19(Suppl 15):435. doi: 10.1186/s12859-018-2420-y (PMC6266932; doi:10.1186/s12859-018-2420-y)
Supplement: Supplementary file 1 — Pseudocode of the Orthologs search. The analysis implements the search for Bidirectional Best Hits considering as input the results of two BLAST based analyses. (ZIP 968 kb) [file 12859_2018_2420_MOESM1_ESM.zip › S8-S1.doc]

**Additional file 1. Pseudocode of the orthologs search.** The analysis implements the search for Bidirectional Best Hits considering as input the results of two BLAST based analyses.

| **Orthologs search** | |
| --- | --- |
| 1: | **procedure** orthologs (BLAST_species1_vs_species2, BLAST_species2_vs_species1) |
| 2: | **for all** species1 query *x_i_* in BLAST_species1_vs_species2 **do** |
| 3: | compute species2 subject *y_j_* with the best score hit |
| 4: | **end for** |
| 5: | **for all** species2 query *y_n_* in BLAST_species2_vs_species1 **do** |
| 6: | compute species1 subject *x_m_* with the best score hit |
| 7: | **end for** |
| 8: | **for all** best score hits *x_i_*,*y_j_* **do** |
| 9: | **for all** best score hits *x_m_* , *y_n_* **do** |
| 10: | **if** *x_i_* =*x_m_* and *y_j_* =*y_n_* **do** |
| 11: | orthologs[*x_i_*][*y_j_*] 🡨 *x_i_* ,*y_j_* |
| 12: | **end if** |
| 13: | **end for** |
| 14: | **end for** |
| 15: | **return** orthologs |
| 16: | **end procedure** |
