# Supplementary material for: Multilevel comparative bioinformatics to investigate evolutionary relationships and specificities in gene annotations: an example for tomato and grapevine
Source: BMC Bioinformatics. 2018 Nov 30;19(Suppl 15):435. doi: 10.1186/s12859-018-2420-y (PMC6266932; doi:10.1186/s12859-018-2420-y)
Supplement: Supplementary file 2 — Pseudocode of the Paralogs search. The analysis implements the identification of paralogs of a given species at a given e-value threshold (k) considering as input the results of a BLAST based analysis. (ZIP 1625 kb) [file 12859_2018_2420_MOESM2_ESM.zip › S8-S2.doc]

**Additional file 2.** **Pseudocode of the paralogs search.** The analysis implements the identification of paralogs of a given species at a given e-value threshold (k) considering as input the results of a BLAST based analysis.

| **Paralogs search** | |
| --- | --- |
| 1: | **procedure** paralogs (BLAST_species1_vs_species1) |
| 2: | pre-defined e-value = k |
| 3: | **for all** Tomato query *x_i_* in BLAST_species1_vs_species1 **do** |
| 4: | compute hit with species1 subject *x_j_* with an e-value ≤ k |
| 5: | **end for** |
| 6: | **for all** hits *x_i_* ,*x_j_* **do** |
| 7: | paralogs[*x_i_*][*x_j_*] 🡨 *x_i_* ,*x_j_* |
| 8: | **end for** |
| 9: | **return** paralogs |
| 10: | **end procedure** |
