# Supplementary material for: Multilevel comparative bioinformatics to investigate evolutionary relationships and specificities in gene annotations: an example for tomato and grapevine
Source: BMC Bioinformatics. 2018 Nov 30;19(Suppl 15):435. doi: 10.1186/s12859-018-2420-y (PMC6266932; doi:10.1186/s12859-018-2420-y)
Supplement: Supplementary file 3 — Pseudocode of the Networks definition. The analysis implements the definition of networks by the NetworkX package (v1.9) considering as input files the results of Orthologs and Paralogs searches. (ZIP 6164 kb) [file 12859_2018_2420_MOESM3_ESM.zip › S8-S3.doc]

**Additional file 3.** **Pseudocode of the Networks definition.** The analysis implements the definition of networks by the NetworkX package (v1.9) considering as input files the results of Orthologs and Paralogs searches.

| **Networks definition** | |
| --- | --- |
| 1: | **procedure** networks (orthologs, paralogs) |
| 2: | **for all** relation *x_i_ , y_j_* in orthologs **do** |
| 3: | node[*x_i_*] 🡨 *x_i_* |
| 4: | node[*y_j_*] 🡨 *y_j_* |
| 5: | edge[*x_i_*][ *y_j_*] 🡨 *x_i_* , *y_j_* |
| 6: | graph[*x_i_*][ *y_j_*] 🡨 node[*x_i_*], node[*y_j_*], edge[*x_i_*][ *y_j_*] |
| 7: | **end for** |
| 8: | **for all** relation *x_i_*, *x_j_* in paralogs **do** |
| 9: | node[*x_i_*] 🡨 *x_i_* |
| 10: | node[*x_j_*] 🡨 *x_j_* |
| 11: | edge[*x_i_*][*x_j_*] 🡨 *x_i_* , *x_j_* |
| 12: | graph[*x_i_*][*x_j_*] 🡨 node[*x_i_*], node[*x_j_*], edge[*x_i_*][*x_j_*] |
| 13: | **end for** |
| 14: | **for all** all nodes and edges in graph **do** |
| 15: | split subgraphs[*z_k_*] of connected components |
| 16: | **end for** |
| 17: | networks[*x_i_*][*y_j_*] 🡨 node[*x_i_*], node[*y_j_*], edge[*x_i_*][*y_j_*], subgraph[*z_k_*] |
| 18: | **return** networks |
| 19: | **end procedure** |
